# High-Blue/Low-Red Mixed Light Modulates Photoperiodic Flowering in Chrysanthemum via Photoreceptor and Sugar Pathways

**DOI:** 10.3390/plants14203151

**Published:** 2025-10-13

**Authors:** Jingli Yang, Zhengyang Cheng, Jinnan Song, Byoung Ryong Jeong

**Affiliations:** 1 Weifang Key Laboratory for Stress Resistance and High Yield Regulation of Horticultural Crops, Shandong Provincial University Laboratory for Protected Horticulture, College of Jia Sixie Agriculture, Weifang University of Science and Technology, Shouguang 262700, China; anne1235606@163.com (Z.C.); jinnansong93@gmail.com (J.S.); 2 Department of Horticulture, Division of Applied Life Science (BK21 Four), Graduate School, Gyeongsang National University, Jinju 52828, Republic of Korea; 3 Division of Horticultural Science, College of Agriculture and Life Sciences, Gyeongsang National University, Jinju 52828, Republic of Korea

**Keywords:** carbohydrate accumulation, florigenic or anti-florigenic genes, high-blue/low-red mixed light, photoperiodic responses, photoreceptors, supplemental or night-interruptional light

## Abstract

Chrysanthemum (*Chrysanthemum morifolium* Ramat.), a typical short-day plant (SDP), relies on photoperiod and light quality signals to regulate flowering and growth. Red light interruptions inhibit its flowering, whereas supplemental blue light can counteract this inhibitory effect. To investigate how “high-blue/low-red” mixed light (RBL) regulates chrysanthemum flowering and growth, we treated ‘Gaya Glory’ plants with 4 h of supplemental or night-interruptional RBL (S-RBL4 or NI-RBL4, 0 or 30 ± 3 μmol m^−2^ s^−1^ PPFD) under 10 h short-day and 13 h long-day conditions (SD10 and LD13; white light, WL; 300 ± 5 μmol m^−2^ s^−1^ PPFD), recorded as SD10, SD10 + S-RBL4, SD10 + NI-RBL4, LD13, LD13 + S-RBL4, and LD13 + NI-RBL4, respectively. Under SD10 conditions, S-RBL4 promoted flowering and enhanced nutritional quality, whereas NI-RBL4 suppressed flowering. Under LD13 conditions, both treatments alleviated flowering inhibition, with S-RBL4 exhibiting a more pronounced inductive effect. Chrysanthemums displayed superior vegetative growth and physiological metabolism under LD13 compared to SD10, as evidenced by higher photosynthetic efficiency, greater carbohydrate accumulation, and more robust stem development. Furthermore, S-RBL4 exerted a stronger regulatory influence than NI-RBL4 on photosynthetic traits, the activities of sugar metabolism-related enzymes, and gene expression. The photoperiodic flowering of chrysanthemum was coordinately regulated by the photoreceptor-mediated and sugar-induced pathways: *CmCRY1* modulated the expression of florigenic genes (*CmFTLs*) and anti-florigenic gene (*CmAFT*) to transmit light signals, while S-RBL4 activated sucrose-responsive flowering genes *CmFTL1/2* through enhanced photosynthesis and carbohydrate accumulation, thereby jointly regulating floral initiation. The anti-florigenic gene *CmTFL1* exhibited dual functionality—its high expression inhibited flowering and promoted lateral branch and leaf growth, but only under sufficient sugar availability, indicating that carbohydrate status modulates its functional activity.

## 1. Introduction

The photoperiod is a key environmental signal that controls plant flowering. Numerous plant species regulate their reproductive cycles by sensing seasonal variations in daylight duration, responding specifically to either long-day (LD) or short-day (SD) conditions [1,2]. The perception of day length is believed to arise from the integration of internal circadian rhythms with external light signals [1,2]. In photoperiodic flowering, light serves two key functions: (1) it resets the circadian clock regulating the timing of clock-controlled genes (CCGs), and (2) it directly influences the activity of these CCGs [3,4]. In *Arabidopsis*, a facultative long-day species (LDS), flowering time is primarily regulated by key photoreceptors including phytochromes, cryptochromes, and members of the ZTL/FKF1/LKP2 protein family [5]. Phytochromes primarily act as photoreceptors for red and far-red light (RL/FRL) and are encoded by a family of five genes, designated *PHYA* through *PHYE*. Phytochrome A (phyA) promotes flowering under FRL [6,7]. In contrast, phyB works with phyD and phyE to inhibit flowering under RL [8,9,10,11,12]. Cryptochrome 1 (cry1) and cryptochrome 2 (cry2) function redundantly to enhance flowering under blue light (BL) conditions [7,13]. Collectively, these photoreceptors modulate flowering time by relaying light signals to the circadian clock and by directly influencing the stability of CONSTANS (CO), a central activator of *FLOWERING LOCUS T* (*FT*) [14,15].

The influence of light quality on flowering regulation differs across plant species. Generally, long-day plants (LDPs) need extended periods of daily light exposure combined with a brief FRL treatment to induce flowering [16,17]. BL enhances flowering in *Cruciferous* species such as *Arabidopsis*, but shows limited effectiveness in other LDP families [16,17]. In contrast to LDPs, short-day plants (SDPs) exhibit lower sensitivity to light quality during the photoperiod and instead depend on prolonged darkness for floral induction. As a result, LDPs and SDPs are commonly categorized as light-responsive and dark-responsive species, respectively [16].

The response of SDPs to light quality during night interruption—defined as a brief light administered within the critical dark period—has been extensively investigated [16]. RL is the most effective in suppressing flowering during such interruptions, and this inhibition can typically be reversed by subsequent exposure to FRL [16]. Phytochromes, photoreceptors sensitive to RL and FRL, have long been recognized as central regulators of photoperiodic responses. Recent genetic evidence confirms their essential role in photoperiodic flowering in rice, a facultative short-day species (SDS) [16]. Complete loss of phytochrome function—as observed in mutants such as *se5* or *phyAphyBphyC*—results in impaired photoperiod sensitivity and precocious flowering under both SD and LD conditions [18,19,20]. In particular, PhyB plays a pivotal role in mediating the flowering delay caused by night-break treatments in rice [21,22]. This occurs through the suppression of *Heading date 3a* (*Hd3a*), the rice ortholog of *FLOWERING LOCUS T* (*FT*), an effect that is abolished in *phyB* mutants [21]. Therefore, phytochromes are not only vital for measuring day length but also indispensable for the inhibition of flowering induced by night interruption in rice.

The influence of light quality during the daily photoperiod on flowering in SDPs has been well documented. For instance, *Pharbitis* seedlings grown under white light (WL) or RL flowered when transferred to inductive dark periods, whereas those cultivated under FRL or BL failed to initiate flowering [23]. In *Xanthium pennsylvanicum*, exposure to RL during the light phase enhanced flowering, while FRL had an inhibitory effect [24]. Similarly, the short-day duckweed strain *Lemna paucicostata* T-101 exhibited characteristic SDP flowering responses under WL or RL conditions but did not flower when initially exposed to BL or FRL [25]. These results collectively indicate that the biologically active form of phytochrome, Pfr, is critical for generating the floral signal during the inductive night, highlighting a Pfr-dependent process in flowering induction.

Chrysanthemum (*Chrysanthemum morifolium* Ramat.) is a major ornamental species and exhibits classic SDP characteristics, initiating flowering when the duration of darkness surpasses a critical threshold [26]. As observed in other SDPs, its floral development is suppressed when the necessary uninterrupted dark period is interrupted by a short pulse of RL, and this suppression can be effectively reversed by subsequent exposure to FRL [26]. The light quality and the ratio of RL to FRL during the daily photoperiod also affect flowering [27,28]. As shown in our previous study, brief exposure to 10~30 μmol m^−2^ s^−1^ PPFD S-BL or NI-BL reverses long-day flowering (LDF) inhibition in chrysanthemum, indicating the involvement of BL-responsive photoreceptors in the flowering response [29]. It was further demonstrated that the activation level of the photosensitive factor is dependent on BL intensity [29]. However, low-intensity (10 μmol m^−2^ s^−1^ PPFD) NI-BL does not induce LDF in the SDP *Kalanchoe blossfeldiana* ‘Rudak’ [30], suggesting species-specific variation in photoperiodic flowering regulation.

Previous studies usually used monochromatic blue light (MBL), which only partially relieved LDF inhibition and failed to overcome residual inhibition from sustained RL receptor activation [29,31,32]. BL also created a trade-off: low intensity inhibited lateral branch development, while high intensity inadequately induced flowering. For example, 30 μmol m^−2^ s^−1^ PPFD suppressed stem elongation in old leaves [32], whereas 40 μmol m^−2^ s^−1^ PPFD improved photosynthetic efficiency but disrupted flowering-related gene expression [29].

This study aims to determine a complex interaction of light signaling between the photoperiod and the processing ways of RBL. The monochromatic white light (MWL) during the daily SD10 and LD13 photoperiods, followed by S-RBL4 or NI-RBL4, regulated flowering and growth differently, with S-RBL4 exhibiting a more pronounced promoting effect. Furthermore, at least two distinct types of signaling pathways—photoreceptor-mediated and sugar-mediated pathways—may be involved in the coordinated control of flowering regulation and vegetative growth.

## 2. Results

### 2.1. Growth and Flowering

**Figure 1:**
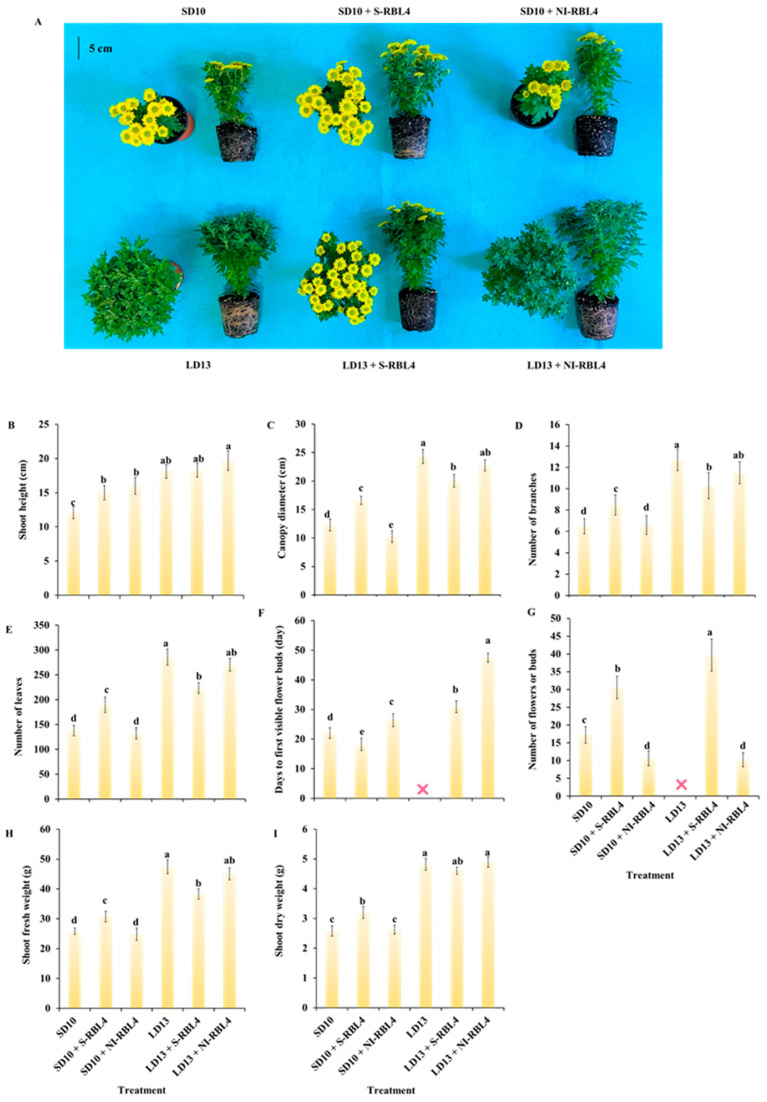
Morphological characteristics (**A**) and growth traits (**B**–**I**) of *Chrysanthemum* ‘Gaya Glory’ following 60 days of cultivation under low-intensity supplemental or night-interruptional lighting with mixed red-blue light. The symbol “❌” denotes treatments that did not induce flowering. The lowercase letters indicate significant separation among treatments by Duncan’s multiple range test at *p* ≤ 0.05 in the same cultivar. Vertical bars represent means ± standard error (n = 6). See Figure 11 for details on light treatments with mixed red-blue light.

The plant height increased with photoperiod duration. Under LD13, plants were 6.0 cm taller than under SD10. Compared to SD10, SD10 + S-RBL4 and SD10 + NI-RBL4 increased height by 2.9 cm and 3.9 cm, respectively. The tallest plants (19.7 cm) occurred under LD13 + NI-RBL4, though S-RBL4 and NI-RBL4 did not significantly promote growth under LD13 conditions (Figure 1A,B).

LD13 conditions promoted crown width, lateral branches, and leaf development more than SD10. LD13 produced the widest crowns and most branches and leaves. Under LD13 conditions, both S-RBL4 and NI-RBL4 suppressed crown expansion and reduced branching, with S-RBL4 showing stronger inhibition. In contrast, under SD10 conditions, S-RBL4 increased crown width and branching, while NI-RBL4 had no effect (Figure 1A,C–E). S-RBL4 also enhanced shoot fresh and dry weights under SD10 but reduced them under LD13 conditions, indicating contrasting effects across photoperiods (Figure 1H,I). Overall, S-RBL4 exerted a stronger influence on growth than NI-RBL4 under both SD10 and LD13 conditions.

In the current study, SD10 was the positive control for flowering, and LD13 the negative control due to insufficient darkness for floral initiation. Under SD10 conditions, S-RBL4 increased flower bud number by 77.26% and advanced emergence by 17.21%, whereas NI-RBL4 reduced buds by 38.11% and delayed flowering by 19.65%. Both S-RBL4 and NI-RBL4 triggered bud formation under LD13 conditions. LD13 + S-RBL4 produced the highest bud count—29.90% more than SD10 + S-RBL4. However, flowering was delayed under LD13 conditions: buds appeared at 30.89 and 44.50 days in LD13 + S-RBL4 and LD13 + NI-RBL4, respectively. By the end of the experiment, buds in LD13 + NI-RBL4 had not opened. No flower buds were observed in the LD13 treatment alone (Figure 1A,F,G).

**Figure 2:**
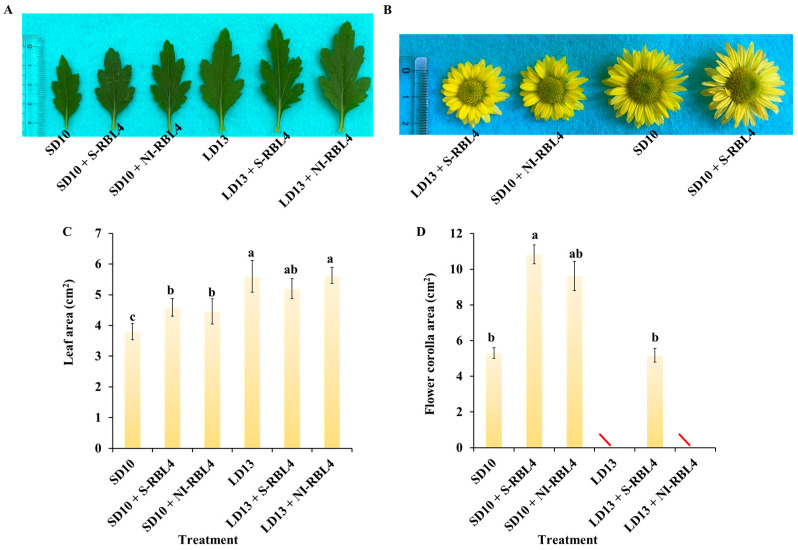
Leaf morphology and size (**A**,**C**), along with flower characteristics (**B**,**D**), of *Chrysanthemum* ‘Gaya Glory’ after 60 days of growth under low-intensity supplemental or night-interruptional lighting with mixed red-blue light. The symbol “\” indicates treatments in which flowering did not occur or flowers did not reach full bloom. The lowercase letters indicate significant separation among treatments by Duncan’s multiple range test at *p* ≤ 0.05 in the same cultivar. Vertical bars represent means ± standard error (n = 6). See Figure 11 for details on light treatments with mixed red-blue light.

The LD13 conditions promoted crown width expansion, lateral branch and leaf development, and leaf size in chrysanthemums more effectively than the SD10 conditions (Figure 2A,C). Compared to the SD10 treatment, both SD10 + S-RBL4 and SD10 + NI-RBL4 significantly increased leaf area, with no significant difference between them. Under LD13 conditions, only LD13 + S-RBL4 slightly reduced leaf area. Under SD10 conditions, the single flower area in SD10 + S-RBL4 was marginally larger than that in the SD10 treatment and significantly greater than that in SD10 + NI-RBL4, showing a 115.28% increase. At full bloom, the flower size in LD13 + S-RBL4 was similar to that in the SD10 + NI-RBL4 treatment, averaging 5.176 cm^2^ and 5.032 cm^2^ respectively (Figure 2B,D).

### 2.2. Stem Anatomical Structures and Stomatal Characteristics

**Figure 3:**
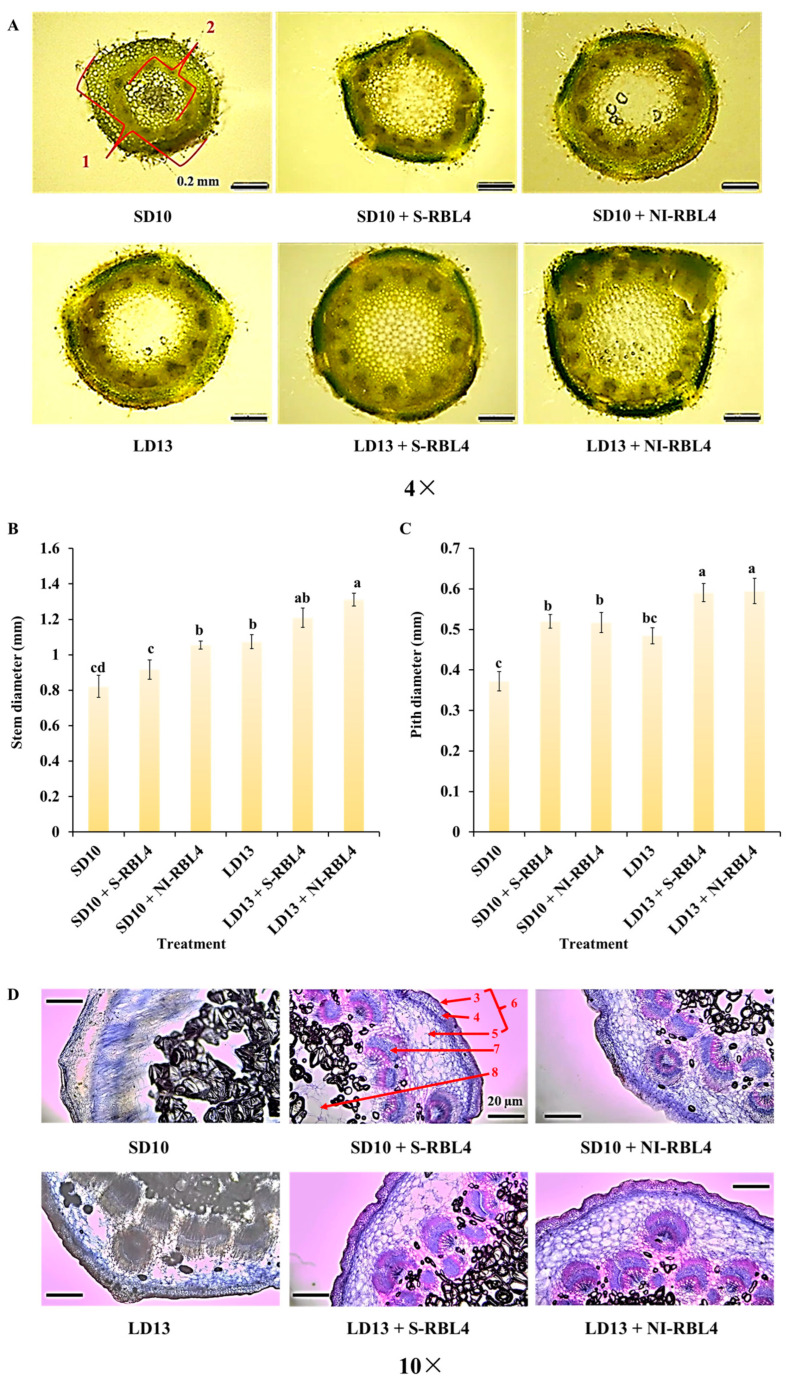
Stem cross sections and associated relative measurements (**A**–**C**), along with microscopic examination of stem microstructures (**D**), in *Chrysanthemum* ‘Gaya Glory’ cultivated under low-intensity supplemental or night-interruptional lighting with mixed red-blue light for 60 days. Labeled components include: 1, stem diameter; 2, pith diameter; 3, cuticle; 4, collenchyma; 5, cortex parenchyma; 6, cortex; 7, vascular bundle; 8, pith. Scale bars in the micrographs represent 0.2 mm in (**A**) and 0.1 mm in (**D**). The lowercase letters indicate significant separation among treatments by Duncan’s multiple range test at *p* ≤ 0.05 in the same cultivar. Vertical bars represent means ± standard error (n = 6). See Figure 11 for details on light treatments with mixed red-blue light.

LD13 conditions promoted better vegetative growth in plants. As shown in Figure 3A–C, chrysanthemums under LD13 conditions had larger stem and pith diameters than those under SD10 conditions. Both S-RBL4 and NI-RBL4 enhanced stem and pith development across photoperiods, with the LD13 + NI-RBL4 treatment yielded the greatest increases—67.22% and 59.95%, respectively, when compared to the smallest measurements. Microscopic analysis (Figure 3D) revealed that both S-RBL4 and NI-RBL4 treatments improved internal stem structure under both photoperiods. Compared to SD10 and LD13 controls, RBL4-treated stems exhibited clearer, more intact, and better-organized cortical cells, with increased parenchyma layers. Collenchyma cells were smaller, more regularly and tightly arranged, and displayed visible cuticle layers on their outer walls. Vascular bundles were well-developed, structurally distinct, and arranged in a ring, clearly separating the cortex from the pith. After safranin and fast green staining, highly lignified cell walls appeared pink, while fibrous walls and other tissues stained bluish-green.

**Figure 4:**
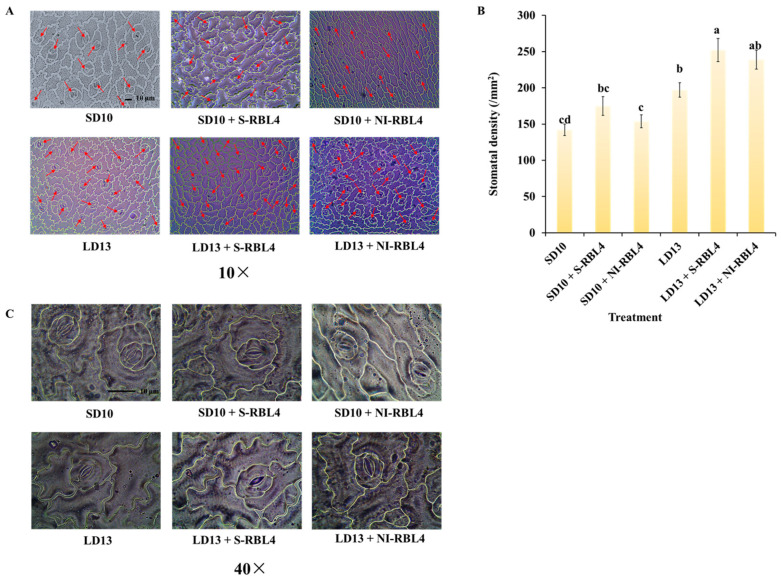
Stomatal density (**A**,**B**) and the status of stomatal aperture (**C**) in *Chrysanthemum* ‘Gaya Glory’ following 60 days of growth under low-intensity supplemental or night-interruptional lighting with mixed red-blue light. The red arrow highlights stomata within the view captured using a 10× objective lens. Scale bars in the micrographs correspond to 10 μm. The lowercase letters indicate significant separation among treatments by Duncan’s multiple range test at *p* ≤ 0.05 in the same cultivar. Vertical bars represent means ± standard error (n = 6). See Figure 11 for details on light treatments with mixed red-blue light.

Stomata are specialized structures in the plant epidermis, primarily on leaf surfaces. This study showed that photoperiod combined with S-RBL4 and NI-RBL4 treatments distinctly affected stomatal density and aperture in chrysanthemum leaves (Figure 4). Stomatal density on the lower epidermis was significantly higher under LD13 than SD10 conditions. Under both SD10 and LD13 conditions, S-RBL4 and NI-RBL4 treatments increased stomatal density, with S-RBL4 having a stronger effect. The highest density occurred with LD13 + S-RBL4, which was 77.46% greater than the SD10 treatment (Figure 4A,B). High-magnification microscopy revealed that both RBL4 variants increased stomatal aperture compared to controls, with S-RBL4 showing the greatest enhancement. As shown in Figure 4C, LD13 + S-RBL4 produced the widest stomatal opening.

### 2.3. Chlorophyll Content

**Figure 5:**
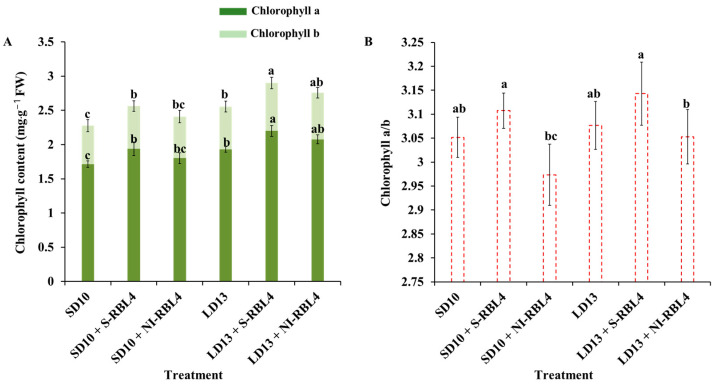
Chlorophyll concentration (**A**) and the chlorophyll a/b ratio (**B**) in *Chrysanthemum* ‘Gaya Glory’ following 60 days of growth under low-intensity supplemental or night-interruptional lighting with mixed red-blue light. Different lowercase letters indicate significant separation among treatments by Duncan’s multiple range test at *p* ≤ 0.05. Vertical bars represent means ± standard error (n = 6). See Figure 11 for details on light treatments with mixed red-blue light.

As shown in Figure 5, the LD13 environments extended photoperiods and promoted vegetative growth in chrysanthemums, leading to higher leaf chlorophyll (Chl) content than SD10 conditions. The LD13 treatment increased Chl a + b by 12.25% compared to SD10. Both S-RBL4 and NI-RBL4 enhanced Chl a and b synthesis under photoperiodic conditions, with S-RBL4 showing a stronger effect. In LD13 + S-RBL4, Chl a increased by 0.271 mg·g^−1^ FW and Chl b by 0.073 mg·g^−1^ FW; in SD10 + S-RBL4, Chl a rose by 0.224 mg·g^−1^ FW and Chl b by 0.062 mg·g^−1^ FW. S-RBL4 significantly increased the Chl a/b ratio relative to SD10 and LD13 treatments, whereas NI-RBL4 tended to reduce it. Nonetheless, the Chl a/b ratio remained close to 3 across all treatments. The LD13 + S-RBL4 treatment achieved the highest levels of Chl a, Chl b, and the Chl a/b ratio.

### 2.4. Photosynthetic and Chlorophyll Fluorescence Parameters

**Figure 6:**
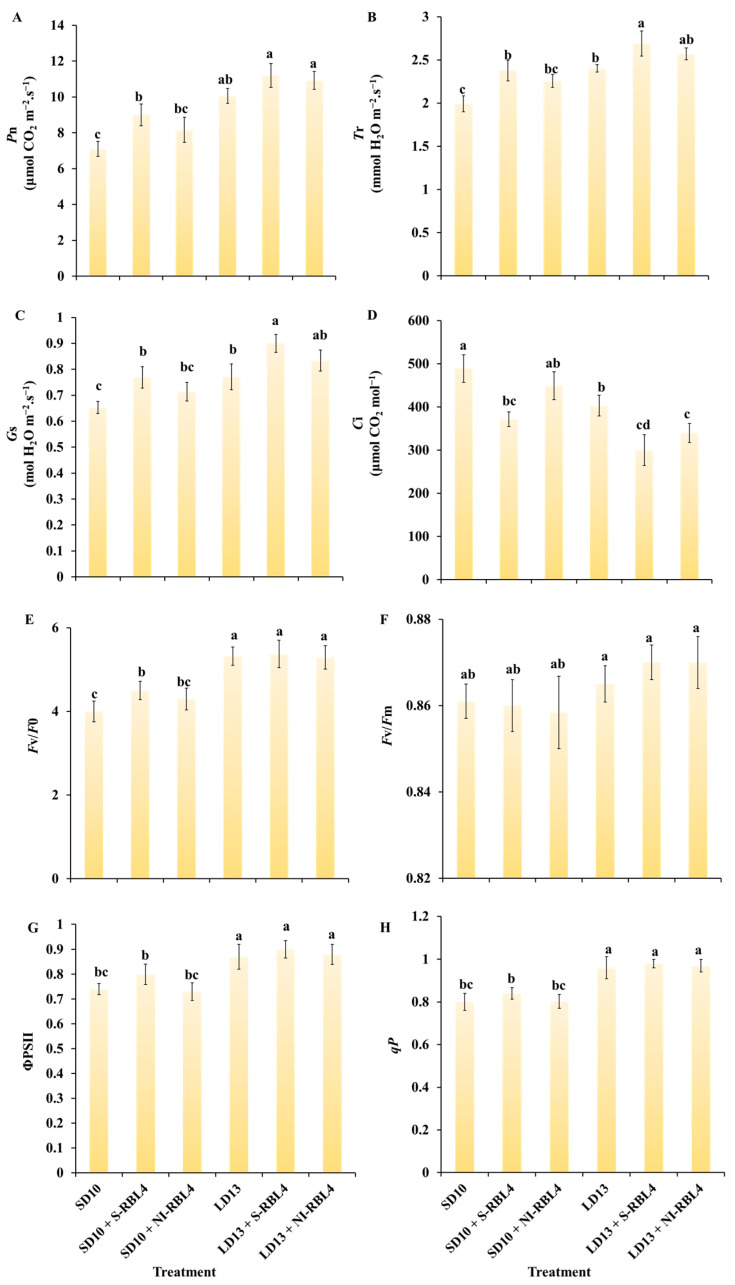
Assessment of photosynthetic parameters (**A**–**D**) and chlorophyll fluorescence characteristics (**E**–**H**) in *Chrysanthemum* ‘Gaya Glory’ after 60 days of cultivation under low-intensity supplemental or night-interruptional lighting with mixed red-blue light. *P*n: net photosynthetic rate; *T*r: transpiration rate; *G*s: stomatal conductance; *C*i: intercellular CO_2_ concentration. *F*v/*F*0: photosystem II (PSII) potential photochemical efficiency; *F*v/*F*m: the maximal PSII quantum yield; ΦPSII: the actual photochemical efficiency in photosystem II; *qP*: the photochemical quenching coefficient. Different lowercase letters indicate significant separation among treatments by Duncan’s multiple range test at *p* ≤ 0.05. Vertical bars represent means ± standard error (n = 6). See Figure 11 for details on light treatments with mixed red-blue light.

Significant correlations were found between photosynthetic parameters and stomatal traits inleaves under different treatments (Figure 4). As shown in Figure 6A–D, net photosynthetic rate (*P*n) was higher under LD13 than SD10 conditions. Plants regulated intercellular CO_2_ concentration (*C*i) by adjusting stomatal aperture or density. Higher CO_2_ availability increased substrate supply for photosynthesis. Increased stomatal density or aperture enhances stomatal conductance (*G*s), facilitating CO_2_ diffusion into mesophyll cells and reducing *C*i. Since CO_2_ is essential for photosynthesis, lower *C*i indicates greater CO_2_ assimilation and thus higher *P*n. Compared to SD10 and LD13 treatments, both S-RBL4 and NI-RBL4 improved photosynthesis-related parameters including *P*n, transpiration rate (*T*r), and *G*s, with S-RBL4 showing a stronger effect. A significant opposite trend was observed between *C*i and *G*s. Consequently, S-RBL4 consistently reduced *C*i under both LD13 and SD10 conditions. The greatest reduction occurred with LD13 + S-RBL4, which achieved the lowest *C*i value—38.65% lower than the highest control.

As shown in Figure 6E–H, photoperiod was a key factor influencing chlorophyll fluorescence parameters, with values under LD13 significantly higher than under SD10 conditions. Under SD10, the SD10 + S-RBL4 treatment increased *F*v/*F*0, ΦPSII, and *qP* to varying degrees. In contrast, under LD13 conditions, neither S-RBL4 nor NI-RBL4 notably affected these parameters.

### 2.5. Carbohydrates and Soluble Protein

**Figure 7:**
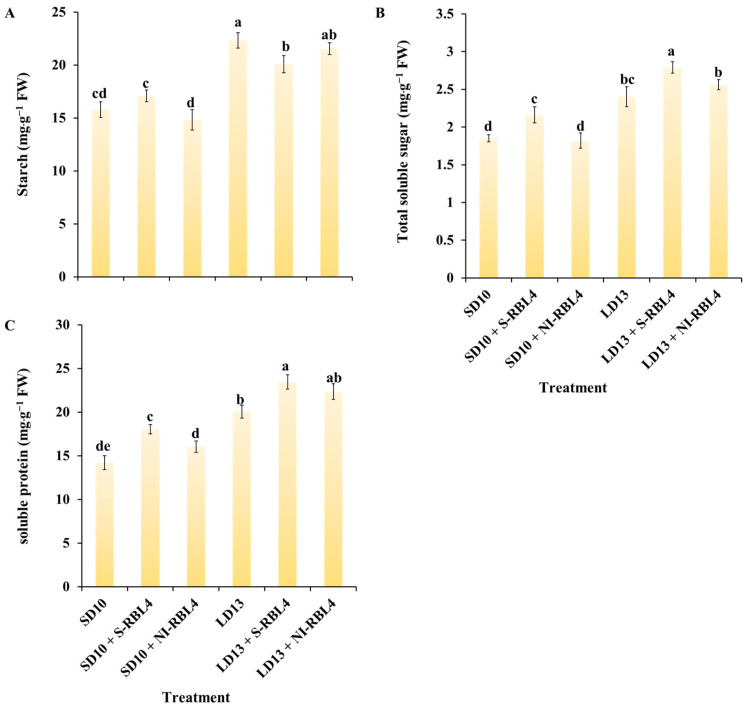
Quantification of carbohydrate levels (**A**,**B**) and total soluble protein content (**C**) in *Chrysanthemum* ‘Gaya Glory’ following 60 days of growth under low-intensity supplemental or night-interruptional lighting with mixed red-blue light. Different lowercase letters indicate significant separation among treatments by Duncan’s multiple range test at *p* ≤ 0.05. Vertical bars represent means ± standard error (n = 6). See Figure 11 for details on light treatments with mixed red-blue light.

The findings showed that LD13 conditions significantly enhanced starch, soluble sugar, and soluble protein accumulation in leaves, with higher concentrations than under SD10 conditions (Figure 7). In the LD13 treatment, starch accumulation increased by 6.55 mg·g^−1^ FW compared to SD10, soluble sugars by 0.55 mg·g^−1^ FW, and soluble proteins by 5.83 mg·g^−1^ FW. Both S-RBL4 and NI-RBL4 significantly affected carbohydrate and soluble protein accumulation regardless of photoperiod. Under SD10 conditions, S-RBL4 increased starch, soluble sugars, and soluble proteins by 1.30, 0.31, and 3.83 mg·g^−1^ FW, respectively. In contrast, NI-RBL4 reduced starch and soluble sugars by 0.95 and 0.03 mg·g^−1^ FW. Under LD13 conditions, both S-RBL4 and NI-RBL4 reduced starch accumulation by 2.25 and 0.78 mg·g^−1^ FW, respectively, but increased soluble sugar and soluble protein levels. The LD13 + S-RBL4 treatment achieved the highest levels of soluble sugars and proteins, while LD13 alone had the highest starch content.

### 2.6. Enzyme Activities Related to Sugar Metabolism

**Figure 8:**
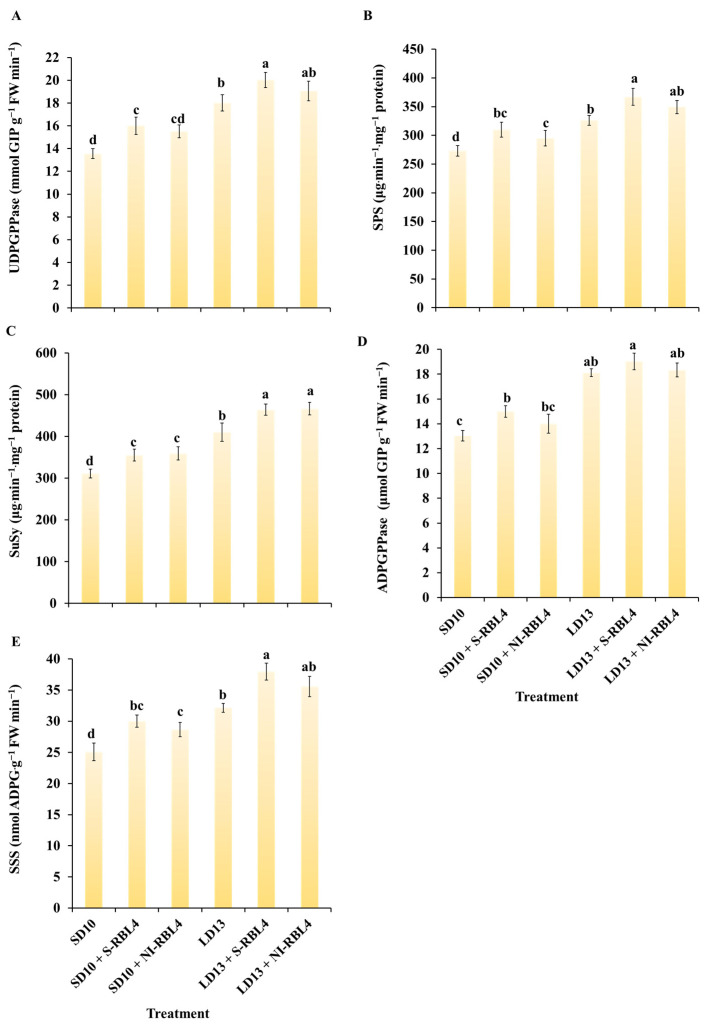
Assessment of enzyme activities related to carbohydrate biosynthesis in *Chrysanthemum* ‘Gaya Glory’ after 60 days of cultivation under low-intensity supplemental or night-interruptional lighting with mixed red-blue light. (**A**) UDGPPase: uridine diphosphate glucose pyrophosphorylase; (**B**) SPS: sucrose phosphate synthase; (**C**) SuSy: sucrose synthase; (**D**) ADPGPPase: adenosine diphosphate glucose pyrophosphorylase; (**E**) SSS: soluble starch synthase. Different lowercase letters indicate significant separation among treatments by Duncan’s multiple range test at *p* ≤ 0.05. Vertical bars represent means ± standard error (n = 6). See Figure 11 for details on light treatments with mixed red-blue light.

As shown in Figure 8, the activities of five key sugar metabolism enzymes—UGDPPase, SPS, SuSy, AGPase, and SSS—were higher under LD13 than SD10 conditions. Across photoperiods, S-RBL4 enhanced these enzyme activities more effectively than NI-RBL4. The LD13 + S-RBL4 treatment yielded the highest activities for all five enzymes among all treatments.

### 2.7. Expression Level of Flowering- or Photoreceptor-Related Genes

**Figure 9:**
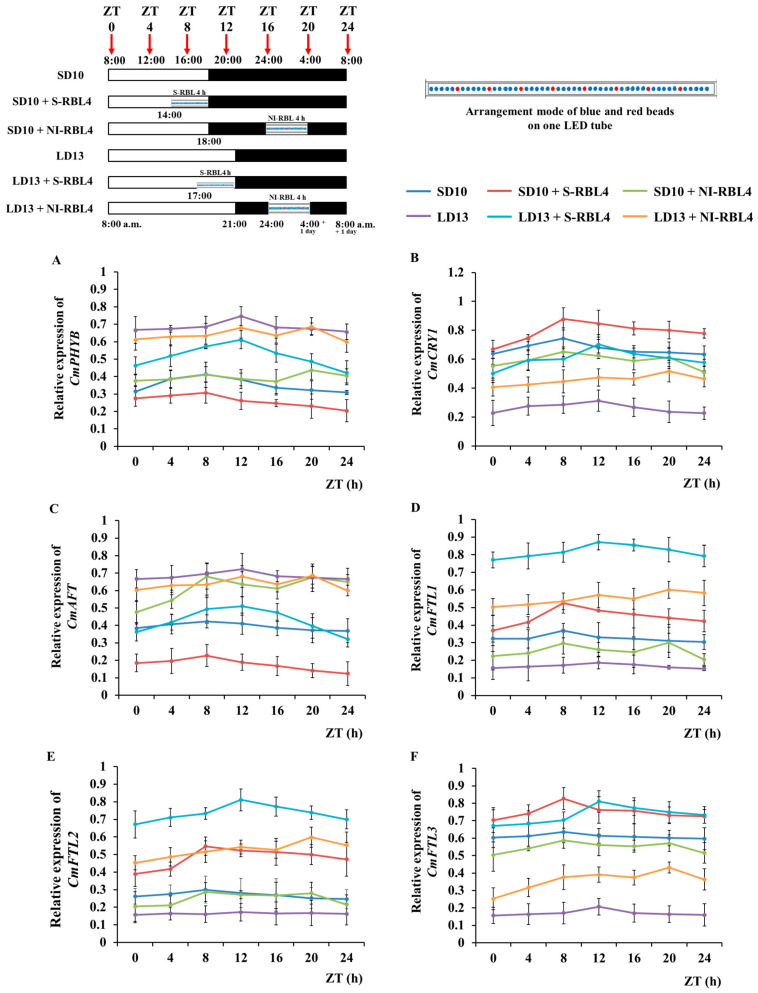
Temporal expression profiles (**A**–**F**) of flowering- and photoreceptor-related genes in *Chrysanthemum* ‘Gaya Glory’ following 7 days of growth under low-intensity supplemental or night-interruptional lighting with mixed red-blue light. The fourth uppermost leaves were collected at 0, 4, 8, 12, 16, 20, and 24 h after light onset (starting at 8:00 a.m.), corresponding to zeitgeber times ZT 0, 4, 8, 12, 16, 20, and 24, respectively, for RNA extraction and RT-qPCR analysis. Gene expression levels were normalized to the reference genes *CmACTIN* and *CmEF1α*, and the highest value in each gene set was scaled to 1 for relative comparison. No statistical significance analysis was conducted; only the overall trend of change in the temporal expression pattern of related genes was presented. Vertical bars indicate the means ± standard error (n = 6). See Figure 11 for details of light treatments with mixed red-blue light.

Chrysanthemum photoperiodic flowering exhibited a clear daily rhythm and was highly responsive to light and dark conditions (Figure 9). After seven days of photoperiodic treatment, the fourth fully expanded leaf from the apex was collected at 0, 4, 8, 12, 16, 20, and 24 h after lights-on (8:00 a.m.). Based on their 24 h expression patterns, the seven genes were grouped into three categories: (1) the red light receptor gene *CmPHYB* and the anti-florigenic gene *CmAFT*; (2) the blue light receptor gene *CmCRY1* and the florigen gene *CmFTL3*; and (3) the LD florigen-*RFT1*-like genes *CmFTL1* and *CmFTL2*.

Under LD13 conditions, *CmPHYB* and *CmAFT* expression peaked first at ZT12 in the LD13, LD13 + S-RBL4, and LD13 + NI-RBL4 treatments, with the highest peak in the non-flowering LD13 treatment. A second peak occurred at ZT20 in the LD13 + NI-RBL4 treatment (end of NI-RBL4), with only a small difference between the two. Under SD10 conditions, both genes peaked first at ZT8 across all three treatments, with maximum expression in SD10 + NI-RBL4, where flowering was suppressed, followed by a slightly lower second peak at ZT20 in the same treatment. Overall, *CmAFT* and *CmPHYB* showed higher expression under LD13 conditions and in treatments that delayed or inhibited flowering.

Regardless of photoperiod, the expression peak times (ZT, h) of *CmCRY1* and *CmFTL3* closely match those of *CmPHYB* and *CmAFT*. Under SD10 conditions, the first peak occurred at ZT8, with a second peak only at ZT20 in the SD10 + NI-RBL4 treatment. Under LD13 conditions, the first peak was at ZT12, and a second peak only at ZT20 in the LD13 + NI-RBL4 treatment. Notably, *CmCRY1* and *CmFTL3* showed higher expression under SD10 conditions or in treatments that promoted flowering—opposite to the pattern of *CmPHYB* and *CmAFT*.

The expression patterns of *CmFTL1* and *CmFTL2* were similar. Under different photoperiod conditions, their peak expression times and trends were largely consistent with those of previously analyzed gene groups. Expression was highest under the flowering-induced LD13 + S-RBL4 treatment, lowest under the non-flowering LD13 treatment, and intermediate under the flowering-inhibited SD10 + NI-RBL4 treatment. Furthermore, *CmPHYB* regulated both *CmFTL3* and *CmAFT* expression, upregulating *CmAFT* but downregulating *CmFTL3*.

**Figure 10:**
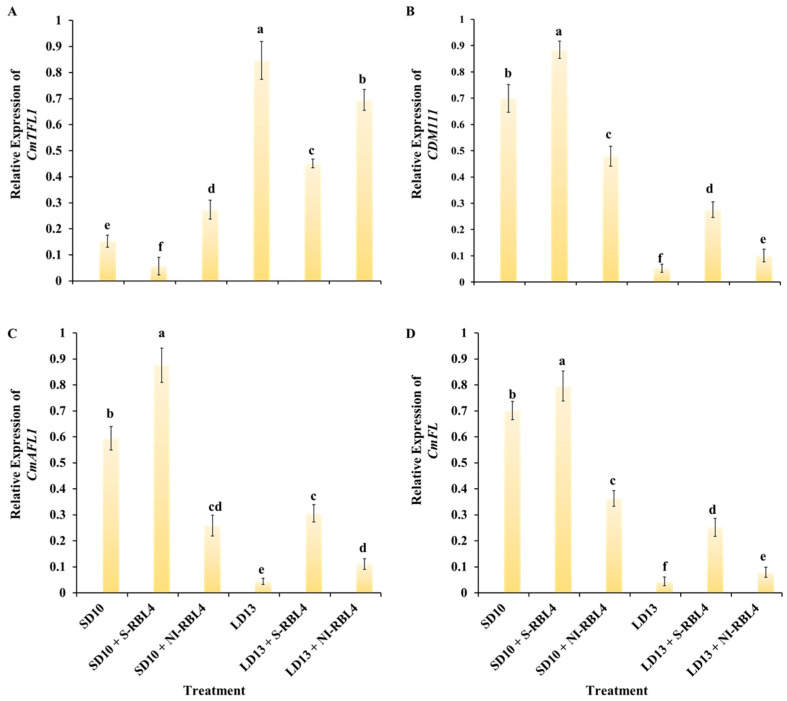
Expression of genes associated with floral development (**A**–**D**) in the shoot apices of *Chrysanthemum* ‘Gaya Glory’ following 7 days of cultivation under low-intensity supplemental or night-interruptional lighting with mixed red-blue light. Shoot apex samples were collected at ZT4 (12:00 p.m.) for RNA extraction and RT-qPCR analysis. Expression levels were normalized to the reference genes *CmACTIN* and *CmEF1α*, and the highest expression value in each dataset was scaled to 1 for relative quantification. Different lowercase letters indicate significant separation among treatments by Duncan’s multiple range test at *p* ≤ 0.05. Vertical bars represent means ± standard error (n = 6). See Figure 11 for details on light treatments with mixed red-blue light.

As shown in Figure 10, after seven days of photoperiodic treatment, the anti-florigenic gene *CmTFL1* was more highly expressed under LD13 than SD10 conditions, particularly in treatments that inhibited flowering or completely blocked floral initiation, such as LD13 and LD13 + NI-RBL4. Regardless of photoperiod, S-RBL4 significantly suppressed *CmTFL1* expression, leading to a marked enhancement in flowering capacity. The floral meristem identity genes *CDM111*, *CmAFL1*, and *CmFL* showed largely opposite patterns to *CmTFL1*: higher under SD10 than LD13 conditions, particularly in SD10 + S-RBL4, but reduced in SD10 + NI-RBL4 treatment. Expression was lower in LD13 + NI-RBL4 and nearly undetectable in non-flowering LD13 treatment. These genes positively correlated with flowering capacity, and their expression levels aligned with the degree of flower induction observed (Figure 1A,G).

## 3. Discussion

### 3.1. Nutritional Growth and Physiological Characteristics in Response to “High-Blue/Low-Red” Mixed Light in Photoperiodic Treatments

The results of our study showed that extending the photoperiod significantly enhanced shoot vegetative growth in *Chrysanthemum* plants. This was supported by increases in plant height (Figure 1B), dry and fresh weight (Figure 1H,I), stem diameter (Figure 3A,B), chlorophyll content (Figure 5), carbohydrate content (Figure 7A,B), and soluble protein content (Figure 7C). Photoperiod serves as a critical environmental cue for seasonal dormancy [33], significantly affecting material production by regulating the duration of leaf absorption and accumulation of photosynthetically active radiation (PAR) [34]. It also acts as a key signaling factor, activating diverse plant signal transduction pathways and thereby influencing assimilate synthesis and allocation through modulation of photosynthetic activity duration in leaves [35,36]. Therefore, under optimal light intensity, extending the photoperiod is generally recommended to promote enhanced vegetative growth in plants [37]. Conversely, under SD conditions, where darkness lasts longer than light, plants typically suppress growth to conserve accumulated carbohydrates [38]. Previous studies on the effects of photoperiod have also confirmed the aforementioned conclusions [39,40]. For example, Zhu Kaiyuan et al. found that extending the photoperiod significantly increased stem height in *Podocarpus macrophylus* (Thunb.) D. D. Don and *Acer palmatum* Thunb., suggesting that photosynthetically derived assimilates are mainly allocated to longitudinal stem growth [40]. In contrast, a shortened photoperiod may reduce dry matter accumulation and inhibit plant height growth. Furthermore, photoperiod regulates branch and leaf development. SDPs remain in the vegetative stage and do not flower under LD conditions [41]. As shown in Figure 1A,D,E, a LD environment greatly promoted lateral branching and leaf production in chrysanthemums.

The rapid rise in fluorescence reflects how the kinetics of redox reactions in the photosynthetic electron transport chain affect chlorophyll fluorescence intensity. This study showed that extending the photoperiod significantly increased the *F*v/*F*0 and *F*v/*F*m values in chrysanthemum plants (Figure 6E,F), indicating that photoperiod variation strongly influenced photosynthetic system functionality. *F*v/*F*m represents the maximum quantum efficiency of PSII in leaves [42], while PIABS comprehensively assesses PSII activity by integrating three key processes: light absorption, excitation energy capture, and electron transport [43]. Additional studies suggest that when plants show low sensitivity to external stresses like drought, *F*0 and *F*m may change in a coordinated way, maintaining a stable *F*v/*F*m value [44], a pattern consistent with this study’s findings. Except for the significant decrease in *F*v/*F*m caused by shortening the photoperiod, the addition of S-RBL4 and NI-RBL4 treatments did not cause notable changes in *F*v/*F*m. Thus, photoperiod shortening likely inhibited leaf photochemical capacity, while S-RBL4 and NI-RBL4 treatments under different photoperiods had little effect on photosynthetic structure functionality. This study further showed that extending the photoperiod significantly increased ΦPSII and *qP* values (Figure 6G,H), indicating that photoperiod extension improved the efficiency and selectivity of photochemical reactions. Yao Ning et al. similarly found that prolonged photoperiod enhances electron transfer from QA to QB within PSII, increasing the PSII electron transport rate [45]. In conclusion, leaf photosynthesis is much more sensitive to photoperiod changes than to short-term, low-intensity supplementary or night-interruption light quality treatments. As the photoperiod lengthens, light energy conversion efficiency and PSII activity increased, the photosystem’s linear electron transport capacity strengthened, and overall photosynthetic assimilation efficiency improved.

Light quality acts as a regulatory signal for seed germination, tissue differentiation, and flower bud formation [46,47]. It regulates hormone levels and enzyme activity through plant photoreceptor activation [48,49], thereby influencing substance synthesis, metabolism, and growth and development [50]. Red light (RL) matches the absorption peaks of plant leaf pigments, promoting cell division and expansion. Blue light (BL) enhances the activity of key enzymes involved in photoreceptor responses, signaling, pigment synthesis, carbon and nitrogen metabolism, chloroplast development, morphogenesis, stomatal movement, photosynthesis, and sugar synthesis [51,52,53,54,55,56,57,58]. Combining red and blue light (RBL) effectively regulates plant growth [59]. In this study, a “high blue–low red” mixed light was used as supplemental or night-interruptional light under different photoperiod conditions. The results showed that both S-RBL4 and NI-RBL4 treatments affected chrysanthemum growth and development to varying degrees. Notably, the S-RBL4 treatment had a significantly stronger promoting effect, as shown by improvements in stem anatomy (Figure 3D), stomatal features (Figure 4), chlorophyll content (Figure 5A), photosynthetic efficiency (Figure 6), organic matter accumulation (Figure 7), and enzyme activities related to sugar synthesis and metabolism (Figure 8).

In a conclusion, both S-RBL4 and NI-RBL4 treatments significantly affected chrysanthemum growth and physiology during photoperiod regulation. Importantly, RBL effects were not independent but interacted with the photoperiod signaling pathway. Additionally, plants show interspecific and intraspecific variations in physiological responses to different photoperiods and light qualities [60,61,62,63,64]. However, the mechanisms underlying the interaction between photoperiod and light quality remain poorly understood, requiring further research to clarify their specific impacts on photosynthesis, growth, and development.

### 3.2. Photoperiodic Flowering in Response to Photoreceptor-Mediated Florigenic and Anti-Florigenic Gene Expression Under “High-Blue/Low-Red” Mixed Light in Photoperiodic Treatments

It is well established that inductive photoperiods stimulate leaves to produce a floral signal called “florigen”. However, it has also been suggested that an anti-florigenic signal from the leaves may regulate photoperiodic flowering, with the right day length suppressed or inactivated this anti-florigen [16,65]. AFT, an anti-florigenic member of the FT/TFL1 protein family, was first discovered in *Chrysanthemum seticuspe*, and strong evidence showed that the CsAFT protein acts as a systemic floral inhibitor—an anti-florigenic signal produced in leaves under non-inductive conditions. Furthermore, studies on the photoperiodic responses of *CsAFT*-RNAi plants supported the key role of the anti-florigenic signal CsAFT in maintaining the vegetative state [66]. Thus, photoperiodic control of florigen synthesis is essential for flowering initiation, and the *CmPHYB*-mediated anti-florigen gene *CmAFT* plays a central role in chrysanthemum’s strict photoperiodic flowering response, ensuring continued vegetative growth under non-inductive conditions (Figure 1 and Figure 9A,C). Chrysanthemum is an obligate SDP that remains vegetative without inductive LD conditions, such as the LD13 treatment used in our study (Figure 1). In contrast, rice (*Oryza sativa*), a facultative SDP, can flower even under non-inductive LD conditions. In rice, two florigen genes, *Hd3a* and *RFT1*, are day length regulated, with RFT1 proposed to act as the LD florigen [67]. In chrysanthemum, *CmFTL1* may function similarly to *RFT1* in rice and serve as an LD florigen gene.

Studies on flowering-related gene expression in leaves improved our understanding of the regulatory mechanisms controlling chrysanthemum flowering. To date, three *FT* orthologues in *Chrysanthemum seticuspe*—*CsFTL1*, *CsFTL2*, and *CsFTL3*—have been identified, with *CsFTL3* having been recognized as a key regulator of photoperiodic flowering [68]. Under inductive SD conditions, *CsFTL3* activates floral identity genes, promoting flowering in the shoot apical meristem (SAM). Moreover, *CsFTL3* overexpression has been shown to trigger flowering in SDPs under LD conditions, indicating its ability to initiate flowering even in non-inductive photoperiods [68].

It could be inferred that under LD photoperiod conditions unfavorable for flowering, the excess *CmFTL3* and upregulated *CmFTL1* worked together, ultimately delaying flowering in the LD13 + NI-RBL4 treatment (Figure 1A,F and Figure 9D,F). *CmFTL3* and the photoreceptor gene *CmCRY1* showed increased expression in both the SD10 + S-RBL4 and LD13 + S-RBL4 treatments. However, their expression was reduced in the LD13 + NI-RBL4 treatment and was nearly absent in the non-flowering LD13 treatment (Figure 9B,F). Previous studies showed that *CmCRY1* up-regulates *CmFTL3*, but the role of RL receptors in this process remained unclear [29]. Here, expression analysis reveals a regulatory cascade: “*CmPHYB*⟶*CmAFT*⟶*CmFTL3*”. S-RBL4 down-regulated *CmPHYB* and suppressed *CmAFT*, thereby relieving repression of *CmFTL3*; NI-RBL4 had the opposite effect (Figure 9A,C,F).

In summary, photoperiodic flowering in chrysanthemum was controlled by photoreceptor-dependent mechanisms and regulated by the coordinated action of florigen and anti-florigen. The balance between these two factors determined the plant’s flowering response to different photoperiods.

### 3.3. Photoperiodic Flowering in Response to the Co-Regulation of Photoperiod- and Sucrose-Mediated Pathways Under “High-Blue/Low-Red” Mixed Light in Photoperiodic Treatment

Sugar signaling plays a key role in various developmental processes, including flowering regulation [69,70,71]. Sucrose is the most common sugar synthesized by plants and is more easily transported due to its greater molecular stability compared to glucose and fructose. In photosensitive plants, exposing a single leaf to an inductive photoperiod quickly increases its sucrose content [72]. In *Arabidopsis*, applying sucrose externally to the aerial parts of dark-grown plants promotes flowering [73]. Sucrose produced by photosynthesis in *Arabidopsis* under LD conditions leads to down-regulation of miR156 [71,73], which in turn increases SQUAMOSA PROMOTER BINDING PROTEIN-LIKE (*SPL*) transcript levels and enhances *FT* expression [74]. However, photoperiod-dependent regulation of florigen alone could not fully explain flowering in chrysanthemum, as the transition from vegetative to reproductive growth is tightly controlled [66].

In the SDP *Chrysanthemum morifolium*, gibberellin signaling and the photoperiod pathway worked together to induce flowering in ‘Floral Yuuka’ under SD conditions. *CmFTL2* transcript levels continued to rise in plants treated with sucrose under both SD and SD + NI conditions [75], indicating that both photoperiod and sucrose signaling regulated *CmFTLs* transcription in ‘Floral Yuuka’. Sugar signaling appeared to be more active under LD conditions [76]. These findings suggested that in ‘Floral Yuuka’ grown under SD conditions, sucrose signaling might play a minor role in floral induction.

This study showed that LD13 combined with S-RBL4 significantly upregulated *CmFTL1/2* expression (Figure 9D,E), an effect dependent on sucrose signaling. Measurements of key sucrose metabolism enzymes (SPS, SuSy, and AGPase; Figure 8), starch and soluble sugar levels (Figure 7), and photosynthetic rate (*P*n increased by 38.65% with S-RBL4; Figure 6) indicated that S-RBL4 enhanced sucrose accumulation by improving photosynthetic efficiency, thereby promoting *CmFTL1/2* expression. In addition, the temporal expression pattern of *CmFTL2* closely matched that of *CmCRY1*, but was opposite to *CmPHYB* (Figure 9A,B,E).

### 3.4. Ornamental Traits of Flowering and Branching in Response to CmTFL1 Under “High-Blue/Low-Red” Mixed Light in Photoperiodic Treatments

According to Gao et al., the *CmTFL1* gene promoted secondary branching in *Arabidopsis* and axillary bud development in *Chrysanthemum*, suggesting that its high expression in the stem supports lateral meristem growth [77]. Similar findings had been observed in other species with homologous *TFL1* genes. For example, in *Lolium perenne* L., the *LpTFL1* gene not only rescued the *tfl1* mutant phenotype but also increased secondary branching and improved vegetative growth, particularly leaf development [78]. Similarly, *AtTFL1* in *Arabidopsis*, *PsTFL1* in *Prunus serotina*, and *LjCEN1* in *Lotus japonicus* have all been shown to enhance branching and leaf production [79,80,81]. Therefore, the *TFL1* gene appeared to have a conserved role in regulating branching and leaf development. Constitutive expression of *CsTFL1* significantly delayed flowering under SD conditions in *Chrysanthemum seticuspe*. The function of *CmTFL1* was further confirmed using five transgenic lines, showing that it influenced flower development in *Chrysanthemum morifolium* [66,82]. Thus, *CmTFL1* acted as an anti-florigenic gene with dual roles: suppressing flowering and promoting vegetative growth, evident as delayed flowering and increased lateral branching and leaf number under high expression.

The study’s findings (Figure 1A,D,E and Figure 10) combined with previous research, showed that *CmTFL1* expression was higher under LD13 than SD10 conditions, supporting its role in flowering inhibition under long photoperiods [32]. Night-interrupted light (NI-RBL4 and NI-BL4) strongly upregulated *CmTFL1*, while supplementary lighting (S-RBL4 and S-BL4) downregulates it. *CmTFL1* expression was inversely correlated with flowering-promoting genes such as *CDM111*, *CmAFL1*, and *CmFL*, and the phenotypic changes reflect its dual role in regulating flowering and vegetative growth [29]. *CmTFL1* expression is tissue-specific, 30 μmol·m^−2^·s^−1^ PPFD monochromatic BL induced a stronger transcriptional response in shoot apical meristems and young leaves, while irradiation of old leaves had little effect on expression [32]. Compared with SD10, SD10 + NI-RBL4 significantly upregulated *CmTFL1* expression and delayed flowering Figure 1F and Figure 10A), but lateral branch and leaf numbers did not increase significantly (Figure 1A,D,E and Figure 10A). This might be attributed to the fact that chrysanthemums primarily regulated flowering in response to night-interrupted light, while the shorter photoperiod limited carbohydrate and nutrient accumulation, thereby restricting the development of additional lateral branches.

This study revealed that *CmTFL1* had dual functions conditional on adequate nutrition. Under LD13 + NI-RBL4, high *CmTFL1* expression and sufficient nutrients inhibited flowering and promoted lateral branching. Under SD10 + NI-RBL4, *CmTFL1* was upregulated but low nutrient levels limit branching, with flowering still inhibited. Prior studies reported either flowering repression [29] or branch promotion [32], but not the nutritional switch. These results explained how chrysanthemum plants allocate resources between flowering and growth.

## 4. Materials and Methods

### 4.1. Experimental Set-Up and Cultivation Conditions

The pot experiment was conducted in a closed-type containerized mini plant factory (770.0 cm long × 250.0 cm wide × 269.5 cm high, Green Industry Co., Ltd., Changwon, Republic of Korea) at Gyeongsang National University, Jinju, Republic of Korea, in early September of 2022. In the plant factory, the temperature, air humidity and the CO_2_ concentration were, respectively, set at 23 °C/18 °C (day/night), 65 ± 5% and 450 μmol·mol^−1^. The ornamental chrysanthemum ‘Gaya Glory’ (*Chrysanthemum morifolium* Ramat.), a qualitative short-day plant (SDP), was obtained from the Flowers Breeding Research Institute, Gyeongnam Agricultural Research & Extension Services, Changwon, Gyeongnam, Republic of Korea. The rooted cuttings with similar morphologies were transplanted from 200-cell plug trays into 10 cm diameter plastic pots filled with commercial BVB medium (Bas Van Buuren Substrates, EN-12580, De Lier, The Netherlands). Older or damaged leaves were removed to ensure that each plant retained approximately ten healthy leaves. The plants were then cultured for 7 days under LD16 conditions (16 h light/8 h dark) using fluorescent lamps at a photosynthetic photon flux density (PPFD) of 270 ± 5 μmol m^−2^ s^−1^. Following this acclimatization period, the plants were exposed to various lighting treatments. Plants were fertilized with multipurpose nutrient solution (macro-elements: Ca^2+^, Mg^2+^, K^+^, NH_4_^+^, NO_3_^−^, SO_4_^2−^, and H_2_PO_4_^−^; micro-elements: B, Cu, Fe, Mn, Mo, and Zn; pH = 6.5) two times per week through root irrigation at 8:00 a.m.

### 4.2. Light Treatments

Light factors in the study were regulated precisely by LED control system (MEF50120 LEDs, More Electronics Co., Ltd., Changwon, Republic of Korea). In this study, the total light intensity of supplemental or night-interruptional mixed red-blue light (RBL) was set at 30 ± 3 μmol m^−2^ s^−1^ PPFD, based on our previous research. This intensity of supplemental or night-interruptional monochromatic blue light (BL) demonstrated a more effective capacity to alleviate long-day flowering inhibition in *Chrysanthemum* [29]. In this study, each light treatment included 24 plants. With six light treatments, the experiment used a total of 144 *Chrysanthemum* ‘Gaya Glory’ plants.

**Figure 11:**
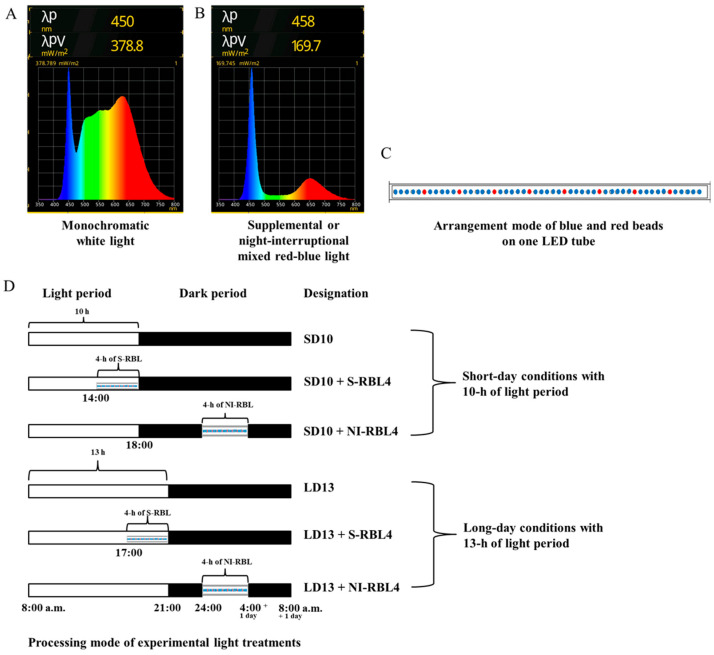
Light Spectrum of (**A**) monochromatic white LEDs and mixed red-blue LEDs (**B**), arrangement mode of red and blue beads on one LED tube (a high proportion of blue light combined with a low proportion of red light, on each LED tube, there is one red LED bead every five blue LED beads; minimum red-to-blue light ratio achievable by this LED system) (**C**), and the processing mode of light treatments (**D**) employed in this study.

As shown in Figure 11, the light treatment was started every day at 8:00 a.m. All tested plants were subjected to the monochromatic white light (WL, 400~800 nm, peak at 450 nm) with intensity of 300 ± 5 μmol m^−2^ s^−1^ PPFD for 10 h (short-day 10 h, SD10) or 13 h (long-day 13 h, LD13); the 4 h of mixed RB LED light (respectively, peak at 650 and 458 nm; red-to-blue ratio: 5:1, which is the minimum ratio achievable by this LED system) with additional total PPFD either of 0 or 30 ± 3 μmol m^−2^ s^−1^ PPFD was used to (1) supplement the WL at the end of the SD10 (SD10 + RBL4) and LD13 (LD13 + RBL4) or (2) provide night-interruption (NI) in the SD10 (SD10 + NI-RBL4) and LD13 (LD13 + NI-RBL4). Due to the obligate SD flowering characteristic of *Chrysanthemum morifolium* Ramat., the plants exposed in SD10 or LD13 conditions without any RBL was set as positive control or negative control, respectively. The light intensity of W and mixed RB LEDs was individually regulated by pulse width modulation (PWM) control, and measured at hte plant canopy level using a LI-250A light quantum meter (LI-COR, Lincoln, NE, USA). The light spectrum was recorded by a spectrometer Lighting Passport Pro (Asensetek, Taiwan, China) with Spectrum Genius Cloud 4.0.1 (New Taipei, Taiwan, China) software. Harvest was carried out on the 60th day after lighting treatment.

### 4.3. Measurement of Plant Growth Indexes

The measurements of the parameters studied were executed in the shoot (whole aboveground parts) of plants at the harvest stage. The measurements included plant height, canopy diameter, stem diameter (middle parts of main stem), number of branches (mature lateral branches on main stem), leaves (fully developed young leaves, length > 1 cm), and flowers (contained both blooming flowers and visible flower buds at the harvest stage), fresh weight (FW), dry weight (DW), leaf area (LA, fully developed young leaves, fourth in the order from the top inflorescences), flower corolla area (the fully bloomed flowers, the same days after the flower buds appeared), and days to the first visible flower buds (the number of days from lighting treatment started to the date when the first flower bud appeared). Plant samples were transferred into a drying oven (Venticell-222, MMM Medcenter Einrichtungen GmbH., Munich, Germany) at 80 °C for 3 days to obtain DW. Every plant’s LA (cm^2^) was measured with an LA meter (CI-202 Laser Area Meter, CID Bio-Science, WA, USA). For each index, measurements were conducted with six biological replicates per treatment, which were randomly selected in a consistent physiological state.

### 4.4. Microscopic Observation of Stem Cross Sections and Stomatal Traits

At harvest, six plants per treatment with consistent physiological states were selected. After cleaning, the middle portion of fresh main stems was sliced to appropriate thickness, and one section from the same position per plant was used. Stem cross sections were mounted on glass slides and observed without staining to measure stem diameter. For detailed observation of internal stem structure, samples were fixed in FAA solution (50% ethanol, 45% paraformaldehyde, 5% glacial acetic acid) at 4 °C for 24 h. Fixed samples were dehydrated through a graded series (twice): 50% ethanol; 50% ethanol + 10% TBA; 50% ethanol + 20% TBA; 50% ethanol + 35% TBA; 50% ethanol + 50% TBA; 25% ethanol + 75% TBA; 25% ethanol + 75% TBA; stained with 0.1% safranin O (45 min each), then cleared in 100% TBA (45 min). Samples were infiltrated overnight with 67% TBA + 33% paraffin (Paraplast X-tra, Kendall, FL, USA), followed by 100% paraffin for 24 h (renewed every 2 h during daytime) at 60 °C, and embedded in paraffin. Sections (10 μm thick) were cut using a rotary microtome (RM2125RT, Leica, Nussloch, Germany), floated on a 42 °C water bath to release compression, and mounted on Superfrost Plus slides (Menzel-Gläser, Braunschweig, Germany). Dried sections were deparaffinized and rehydrated through a graded ethanol series [83]. Additional staining included 1% safranin O in 50% ethanol (3 h) and 0.5% methyl green (45 min) [84]. After washing and dehydration, sections were permanently mounted with Canada balsam under coverslips for microscopic observation.

A commonly used method for analyzing stomatal traits is the nail polish (NP) method. On the day of harvest, under sunny conditions from 10:00 to 12:00 in the morning, six plants per treatment with consistent physiological states were selected, and one mature and healthy leaf per plant from the same position was sampled. Fresh leaves were immediately placed in an ice box at 5 °C, with sectioning and imaging completed within one hour. A thin layer of nail polish (Eco. Top Coat, Innisfree, Republic of Korea) was applied to the abaxial leaf surface, avoiding the main vein, and allowed to dry for 5 min. The dried film was carefully peeled off using clear tape (Crystal Clear Office Tapes, Winc, Sydney, Australia) and mounted on a microscope slide. Stem cross sections and leaf stomatal imprints were observed using an optical microscope (ECLIPSE Ci-L, Nikon, Tokyo, Japan) equipped with a DS-Ri1 color camera and 4×, 10×, or 40× objectives. Images were captured using ImageJ (64-bit Java 1.8.0_172, National Institutes of Health, Bethesda, MD, USA). Stomatal density was determined following Sack and Buckley [85].

### 4.5. Measurement of Photosynthetic and Chlorophyll Fluorescence Parameters

After 60 days of lighting treatment, from 9:00 a.m. to 10:30 a.m., leaf photosynthetic parameters (net photosynthetic rate (*P*n), transpiration rate (*T*r), stomatal conductance (*G*s), and intercellular CO_2_ concentration (*C*i)) were measured non-destructively with a portable photosynthesis system (TARGAS-1, PLC5, LED, PP Systems, Inc., 110 Haverhill Rd, Suite 301 Amesbury, MA 01913, USA) in the closed-type containerized mini plant factory.

The leaf chlorophyll fluorescence measurements were conducted using a Fluor Pen FP 100 (Photon Systems Instruments, PSI, Drásov, Czech Republic). Leaves were dark-adapted with a leaf clip for 30 min, then a 0.6 s saturating light pulse (3450 μmol·m^−2^·s^−1^ PPFD) was given to obtain the maximal fluorescence (*F*m) and minimal fluorescence (*F*0). Then, the leaf was light-adapted with 5 min continuous actinic light (300 ± 5 μmol m^−2^ s^−1^ PPFD, 20~23 °C, CO_2_ concentration 450 μmol·mol^−1^) with saturating pulses every 25 s, after that, the maximum light-adapted fluorescence (*F*m′) and steady-state fluorescence (*F*s) were recorded. The actual photochemical efficiency in photosystem II (ΦPSII = (*F*m′ − *F*s)/*F*m′). The maximal PSII quantum yield (*F*v/*F*m) was calculated as *F*v/*F*m = (*F*m − *F*0)/*F*m [86]. The actinic light was turned off and a far-red pulse was applied to obtain minimal fluorescence after the PSI excitation (*F*0′). The photochemical efficiency of PSII (*F*v′/*F*m′) was calculated as *F*v′/*F*m′ = (*F*m′ − *F*s)/*F*m′. Moreover, the photochemical quenching coefficient (*qP*) was calculated as *qP* = (*F*m′ − *F*s)/(*F*m′ − *F*0′) [87]. For each photosynthetic and chlorophyll fluorescence parameter, six plants per treatment with consistent physiological states were selected, and the fourth fully developed leaf from the top inflorescence was analyzed.

### 4.6. Determination of Chlorophyll Content

On the day of harvest, at 5 p.m., the total chlorophyll (Chl) content was determined from fresh medium-aged leaves with excluded the edges and veins of leaves (fourth leaf from the top). Tissues of fresh leaves (0.2 g) were cut, ground well, suspended in 5 mL of 95% (*v*/*v*) ethanol and filtered. The filtrate was made up to 25 mL by adding 95% (*v*/*v*) ethanol. Absorbance of the filtered solution for Chl a and Chl b at 665 nm, 649 nm, respectively, was measured using a Libra S22 UV spectrophotometer (Biochrom Ltd., Cambridge, UK), while the Chl content was determined using Equations (1)–(3), following [88]:

Chl a (mg g^−1^ FW) = (13.95 OD_665_ − 6.88 OD_649_) V/200 W (1)

Chl b (mg g^−1^ FW) = (24.96 OD_649_ − 7.32 OD_665_) V/200 W (2)

Chl a/b = Chl a/Chl b(3)

where Chl a is chlorophyll a, Chl b is chlorophyll b, V is volume (25 mL), and W is sample fresh weight (0.2 g). Measurements were conducted with six biological replicates per treatment.

### 4.7. Sample Preparation for Biochemical Analyses and Total RNA Extraction

At the harvest stage, the fully developed young leaves were collected, frozen in liquid nitrogen, and then stocked in the −80 °C conditions. Afterward, they were pulverized (MM400, Retsch, Haan, Germany), and the material was used for further analyses. In addition, for each biochemical index (soluble sugar, starch, soluble protein, and some enzyme activities), RNA extraction, and quantitative RT-PCR (qRT-PCR), six plants per treatment with consistent physiological states were selected.

### 4.8. Determination of Carbohydrates and Soluble Protein

For soluble sugar determination, 0.5 g of fresh sample was mixed with 10 mL distilled water and extracted in a boiling water bath for 30 min (twice). After filtration, the filtrate was diluted to 25 mL with distilled water. A 0.5 mL aliquot of the extract was mixed with 1.5 mL distilled water, 0.5 mL anthrone ethyl acetate reagent, and 5 mL concentrated sulfuric acid, shaken thoroughly, and immediately incubated in a boiling water bath for 1 min. After natural cooling, absorbance was measured at 630 nm using a Libra S22 UV spectrophotometer.

For starch determination, 1.0 g of fresh sample was mixed with 5 mL of 80% (*v*/*v*) ethanol and extracted in an 80 °C water bath for 30 min. After centrifugation at 12,000 × g for 10 min (twice), the precipitate was resuspended in 3 mL distilled water and boiled for 15 min to gelatinize starch. Following cooling, 2 mL of 30% (*v*/*v*) HClO_4_ was added and the mixture agitated. The solution was diluted to 10 mL with distilled water, then centrifuged again at 12,000× *g* for 10 min (twice), and the supernatant collected. Starch content in the supernatant was quantified using the sulfuric acid anthrone method at 485 nm with a Libra S22 UV spectrophotometer [89].

For soluble protein determination, 0.2 g of fresh sample was homogenized in 50 mM PBS (pH 7.0) containing 1 mM EDTA, 1 mM polyvinylpyrrolidone, and 0.05% (*v*/*v*) Triton X-100. The mixture was centrifuged at 12,000× *g* for 20 min at 4 °C, and the supernatant was used for absorbance measurement at 590 nm with a Libra S22 UV spectrophotometer. Total protein content was quantified using the Bradford method [90].

### 4.9. Determination of Enzyme Activities Related to Sugar Metabolism

The total protein solution obtained from the previous step was used to analyze the enzymatic activities and measured through a Libra S22 UV spectrophotometer.

The sucrose synthase (SuSy) and sucrose phosphate synthase (SPS) were determined in a 1 mL reaction mixture containing a 500 μL enzyme extract at 34 °C for 1 h. 300 μL KOH (30% (*v*/*v*)) was added to this mixture and was then placed in a water bath at 100 °C for 10 min, after which it was gradually cooled to room temperature. The mixture was subjected to incubation at 40 °C for 20 min after a 200 μL anthrone–sulfuric acid solution (0.15% (*v*/*v*)) was applied and the enhancement of wavelength at 620 nm was monitored.

Moreover, the activities of soluble starch synthase (SSS), adenosine diphosphate glucose pyro-phosphorylase (ADPGPPase) and uridine diphosphate glucose pyro-phosphorylase (UDGPPase) were measured according to the protocol described by Doehlert et al. and Liang et al. [91,92].

### 4.10. Verification by Real-Time Quantitative PCR

RNA was extracted using the RNeasy Plant Mini Kit (Takara Bio Inc., Tokyo, Japan) according to the manufacturer’s instructions. Reverse transcription of cDNA was performed using PrimeScript^®^ Reverse Transcriptase (Takara Bio Inc., Tokyo, Japan). The cDNA was diluted 10-fold, and 5 μL was used in 15-μL qRT-PCR reactions with SYBR Premix Ex Taq™ II (Takara Bio Inc., Tokyo, Japan), performed in a Roche Light Cycler 96 real-time fluorescence quantitative PCR instrument (Roche, Basel, Switzerland). The 2^−∆∆Ct^ method [93] was used to determine the relative expression levels of each target gene. The chrysanthemum homologues of *Arabidopsis* were written as “Cm + gene” in our study. The *FT*-like genes (*CmFTL1*, *CmFTL2*, and *CmFTL3*) [75,94], the anti-florigenic *FT/TFL1* family gene *CmAFT* [95], and two photoreceptor genes—Phytochrome B (*CmPHYB*) and Cryptochrome 1 (*CmCRY1*) [94,96] were selected to explore the temporal expression patterns of flowering- or photoreceptor-related genes in chrysanthemums. The anti-florigenic *TFL1/CEN*-like gene *CmTFL1* [66] and three floral meristem identity genes—*APETALA1* (*CDM111*), *FRUITFULL* (*CmAFL1*), and *LEAFY* (*CmFL*) [97,98]—analyzed in shoot apex tissues were used to study the expression patterns of floral formation-related genes. Data were averagely normalized against the expression of two reference genes, *CmACTIN* and *CmEF1α* (elongation factor 1α) [94,99]. All the target genes’ primers are listed in Table 1.

### 4.11. Statistical Analysis

In our study, all plants were randomly sampled. The data were processed, plotted, and statistically analyzed in Excel 2016 and DPS software package 19.05 (DPS for Windows, 2009). Significant differences among the treatments were assessed by an analysis of variance (ANOVA), followed by Duncan’s multiple range test at a probability (*p*) ≤ 0.05 with a statistical program (SAS, Statistical Analysis System, V. 9.1, Cary, NC, USA). All experimental assays were conducted on six plants per treatment with consistent physiological states and are presented as mean ± standard error.

## 5. Conclusions

The current study investigated the balance between growth and photoperiodic flowering under S-RBL and NI-RBL conditions in SDP chrysanthemum. It not only maintained the efficient flowering-inductive capacity of BL but also achieved the coordinated control of flowering regulation and vegetative growth through low-intensity RL supplementation. Under SD10, S-RBL4 upregulated the florigenic gene *CmFTL3* and floral identity genes (*CDM111*, *CmAFL1*, *CmFL*), while NI-RBL4 suppressed them. Under LD13, both treatments enhanced these genes, with S-RBL4 showing a stronger effect. In LD13 + S-RBL4, the long-day-responsive florigenic genes *CmFTL1* and *CmFTL2*—regulated by photoperiod and sucrose signaling—were strongly upregulated. In contrast, the anti-florigenic genes *CmAFT* and *CmTFL1* were elevated under flowering-inhibitory conditions like SD10 + NI-RBL4 and LD13. The blue light receptor gene *CmCRY1* was upregulated in flowering-promoting treatments (SD10 + S-RBL4, LD13 + S-RBL4), whereas the red light receptor gene *CmPHYB* increased under suppressive conditions and correlated positively with *CmAFT* and negatively with *CmFTL3*. S-RBL4 and NI-RBL4 (blue-to-red ratio, 5:1) cooperatively regulated chrysanthemum flowering via the photoreceptor-mediated pathway (involving *CmPHYB* and *CmCRY1*) and sucrose signaling (mediated by *CmFTL1/2*). The anti-florigenic gene *CmTFL1* had dual roles: high expression suppressed flowering but promoted lateral branching and leaf growth when carbohydrates are sufficient, indicating that sugar status modulated its function and influenced resource allocation between flowering and growth.

## Figures and Tables

**Table 1:** The information about primers used for qRT-PCR.

Gene	Accession Number	Forward Primer (5′ to 3′)	Reverse Primer (5′ to 3′)	Classification of Gene Functions
*CmACTIN*	AB205087	GATGACGCAGATCATGTTCG	AGCATGTGGAAGTGCATACC	Reference genes
*CmEF1α*	AB548817	CTTGTTGCTTGATGACTGTGG	CTTGTTGCTTGATGACTGTGG	
*CmCRY1*	NM-116961	CGTAAGGGATCACCGAGTAAAG	CTTTTAGGTGGGAGTTGTGGAG	Photoreceptor genes
*CmPHYB*	AB733630	TCCAAGAGGGTCATTTGGAG	ACCTGGCTAACCACAGCATC	
*CmAFT*	AB839766	CAAGCAAAAAGCAAGGCAATCA	CAACCGGTAACCCCAAGTCATT	Anti-florigenic FT/TFL1 family *TFL1*/*CEN*/*BFT*-like gene
*CmFTL1*	AB679270	AATCGTGTGCTATGAGAGCC	GCTTGTAACGTCCTCTTCATGC	*FT*-like genes
*CmFTL2*	AB679271	ATGTGTTATTCCGGCAATTGGGTCG	AAATATGCATTTGTAACGTCATGTG	
*CmFTL3*	AB679272	GGGAAAGTGGATTTGGTGGACG	GTCTTACAATTTGGTACTGTCG	
*CmTFL1*	AB839767	CCATCATCAAGGCACAATTTCA	TTTCCCTTTGGCAGTTGAAGAA	Anti-florigenic *TFL1*/*CEN*-like gene; specifically highly expressed at the shoot apex
*CDM111*	AY173054	GGTCTCAAGAATATTCGCAC	TCATTAGTCATCCCATCAGC	Well-characterized floral meristem identity genes *APETALA1* (*CDM111*), *FRUITFULL* (*CmAFL1*), and *LEAFY* (*CmFL*); specifically highly expressed at the shoot apex
*CmAFL1*	AB451218	CAAGCTCAACCATCAATAGTC	TGCAGCACATGAACGAGTAG	
*CmFL*	AB451217	CATTGATGCCATATTTAACTC	ACACGGATCATTCATTGTATA	

## Data Availability

No new data were created or analyzed in this study. Data sharing is not applicable to this article.
